# Floral Trait Preferences of Three Common wild Bee Species

**DOI:** 10.3390/insects15060427

**Published:** 2024-06-06

**Authors:** Kim C. Heuel, Tim A. Haßlberger, Manfred Ayasse, Hannah Burger

**Affiliations:** Institute for Evolutionary Ecology and Conservation Genomics, University of Ulm, 89077 Ulm, Germanymanfred.ayasse@uni-ulm.de (M.A.); info@hannah-burger.de (H.B.)

**Keywords:** floral color, flower size, floral scent, behavior, *Lasioglossum villosulum*, *Bombus terrestris*, *Osmia bicornis*

## Abstract

**Simple Summary:**

Many plants depend on pollination by bees, whereas bees depend on flowers as food sources. Bees use a variety of floral cues such as the color, scent, or shape of the flower to find host plants. The preferred cues of honeybees and bumblebees are well studied, but the preferences of other bee species are almost unknown. Thus, we have performed behavioral experiments with artificial flowers to test whether three common bee species of the genera *Lasioglossum*, *Bombus*, and *Osmia* are attracted by the same or different floral cues. Our experiments showed consistent behaviors across species in experiments testing flower sizes and scent mixtures that differed in compound richness and identities. The color hue experiments, however, revealed different preferences that were probably influenced by previous foraging experience. Within colors, bee species preferred mostly intense colors that formed a high contrast to background colors. A high attractiveness of floral cues enables bees to effectively find foraging plants. With this study, we learned more about flower choice in bee species that are not used as model organisms but are important for pollination.

**Abstract:**

The interaction between bees and flowering plants is mediated by floral cues that enable bees to find foraging plants. We tested floral cue preferences among three common wild bee species: *Lasioglossum villosulum*, *Osmia bicornis*, and *Bombus terrestris*. Preferences are well studied in eusocial bees but almost unknown in solitary or non-eusocial generalist bee species. Using standardized artificial flowers altered in single cues, we tested preferences for color hue, achromatic contrast, scent complexity, corolla size, and flower depth. We found common attractive cues among all tested bees. Intensively colored flowers and large floral displays were highly attractive. No preferences were observed in scent complexity experiments, and the number of volatiles did not influence the behavior of bees. Differing preferences were found for color hue. The specific behaviors were probably influenced by foraging experience and depended on the flower choice preferences of the tested bee species. In experiments testing different flower depths of reward presentation, the bees chose flat flowers that afforded low energy costs. The results reveal that generalist wild bee species other than well-studied honeybees and bumblebees show strong preferences for distinct floral cues to find potential host plants. The diverse preferences of wild bees ensure the pollination of various flowering plants.

## 1. Introduction

Bees and melittophilic plants have evolved in close association: plants depend on bees to transfer pollen in order to reproduce, and bees depend on floral rewards such as pollen and nectar as food resources [1]. The approximately 600 bee species in Germany [2] markedly differ regarding their phenology, nutritional needs, behavioral preferences, and lifestyles. Bee species forage on a variety of plant species and restrict their foraging based mainly on nectar and pollen properties, morphological barriers, and the abundance of plants [3]. Many bee species are specialized to specific plant taxa, whereas others are generalist flower visitors and collect pollen from a broad plant range [4].

The interaction between bees and their host plants is mediated by floral cues that enable bees to identify and relocate host plants. Yet, bees do not only need to optimize the recognition and handling of certain plant species but also need to be able to identify potential new host plants when flower availability changes during their lifespan. Floral cues appear as multimodal stimuli that are perceived by the various senses of the bees. The complex interplay of olfactory, visual, and haptic features results in a complex task for the bee when interacting with a flower [5]. Bees are able to associate rewards not only with single cues but also with several cues at once, and they can transfer learned cue properties [5,6]. This multimodality results in a combination of floral cues being manifold across different plant species. Accordingly, the behavioral preferences for floral cues of different bee species can be also manifold and different bee species may show different attraction patterns to different cues [7,8]. Whereas olfactory and visual cues have been studied for many decades [9,10,11], behavioral studies that compare floral cue preferences among different bee species remain rare (but see, e.g., [12]). Most experiments examining the floral cue preference of bees have been performed either with widespread model organisms such as *Apis mellifera* and *Bombus terrestris* [5,13,14] or in highly specialized bee–plant interactions [15,16,17]. Studies on generalist solitary and other non-eusocial bee species are rare. This is a crucial gap as such species play a crucial role in pollinator–plant interactions. For example, *Lasioglossum* spp. often dominate wild bee communities and their social behavior is well studied [18,19] but have been neglected in studies of floral cue preference (except the study series on Australian *Lasioglossum* sp. by Howard [20,21,22]). Alongside, *Osmia bicornis* bees, that are commercially used as pollinators of orchard trees and are well studied regarding their pollination efficiency [23,24], are also rarely tested for their floral cue preferences (but see, e.g., [25,26]). Olfactory signals often mediate interactions between flowers and their visitors, and floral bouquets of volatile organic compounds (VOCs) are under strong natural selection by pollinators [27]. The flowers of entomophilous species are often intensely scented and emit a high compound richness (scent complexity) [28]. Melittophilous species normally emit VOCs of various chemical classes such as terpenes (e.g., mono- or sesquiterpenes), benzenoids, or alcohols [29,30], which are perceived at different sensitivities by bees [31,32]. The information transmitted can be manifold and might include information about the reward status of a flower, species-recognition cues regarding defense compounds against floral antagonists, or intra-species communication of the stress-levels of the plants [33,34,35,36]. For this reason, plants often emit complex scent bouquets with different volatiles having different functions [37]. Thus, complex cues enable the simultaneous transmission of a wide range of information. Indeed, complex displays of scent and color cues have been shown to reduce a pollinator’s uncertainty about the floral signal [38].

Other than floral scent, floral color is the most conspicuous floral cue. In some systems, color cues can be even more important for flower choice than floral scent, as shown in the interaction between solitary and honeybees with *Wahlenbergia* spp. (Campanulaceae) plants [12]. Color signals of flowers have various perceptual characteristics, such as hue and contrast that influence bee behaviors, and are often difficult to disentangle from one another (as reviewed in [3]). The dominant wavelength of floral colors is perceived as hue and bees can show preferences for distinct colors, e.g., a preference for the blue colors of *Hoplitis adunca* enabling the specialized bees to find their bluish-colored host flowers [16]. The achromatic contrast between the flowers and their background, e.g., leaf vegetation, is perceived by the green receptor and increases the detectability of the flower [39]. The importance of this contrast has been studied intensively, with strong contrasts being known to be largely preferred [40]. Yet the preferences for certain color properties have been studied mostly in *A. mellifera* and *Bombus* spp. [3,41,42] and studies on other wild bee species are still scarce (but see [12,16]).

The detectability of flowers also depends on the flower size, with large displays being initially perceived at a larger distance by an approaching bee [43]. For example, the composite flower heads of *Asteraceae* species consist of several hundred small flat florets together forming a larger display size. Not only the flower size, but also the flower depth influences bee visitors [44]. Whereas morphologies with a deep spur can be exploited efficiently by long-tongued visitors, the same bees can face problems when exploiting flat flowers [45,46,47].

In this study, we have tested the preferences for various floral cues in three wild bee species in Germany: *Lasioglossum villosulum*, *Osmia bicornis*, and *Bombus terrestris*. The goal of our study has been to investigate whether certain floral cues are generally preferred across bees of different species and foraging experience. The chosen bee species represent a broad range of body sizes, social lifestyles, phenologies, habitat preferences, and floral preferences. *Bombus terrestris* is a eusocial bee and generalist forager and is commonly used for crop pollination. *Osmia bicornis* lives a solitary life but often colonizes nesting aids in large individual numbers. This species is active in the spring/early season and is also commercially used in orchard pollination. *Lasioglossum villosulum* bees have not been commercially used so far. They have small body sizes, live communally in aggregations of ground nests, and have a spring and a summer generation [48]. Due to the different phenologies and behaviors of the bee species, the individual bees differed in their foraging experience when experiments were performed. We have performed bioassays with artificial flowers resembling the different floral cues of the common herb *Anthemis tinctoria*, a highly attractive host plant for many bee species [49]. Different floral traits were presented in two-choice assays with opposing floral cues to reveal preferences for yellow versus blue, colors with a high versus low achromatic contrast, smaller versus larger flower sizes, and floral scent bouquets with simple versus complex VOC mixtures. We have further tested, by means of electroantennography (EAG), the antennal detectability of the VOCs used in the experiments and investigated whether sugar water consumption in rewarding artificial flowers is driven by flower depth.

## 2. Materials and Methods

**Figure 1:**
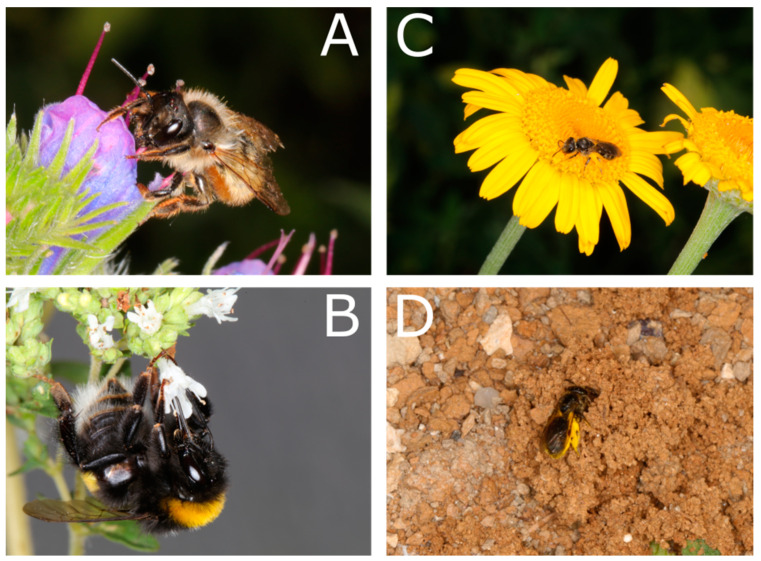
Study organisms (**A**–**D**). (**A**) *Osmia bicornis*, (**B**) *Bombus terrestris*, (**C**) Halictidae bee on *Anthemis tinctoria*, and (**D**) pollen-loaded *Lasioglossum villosulum* returning to nest.

**Figure 2:**
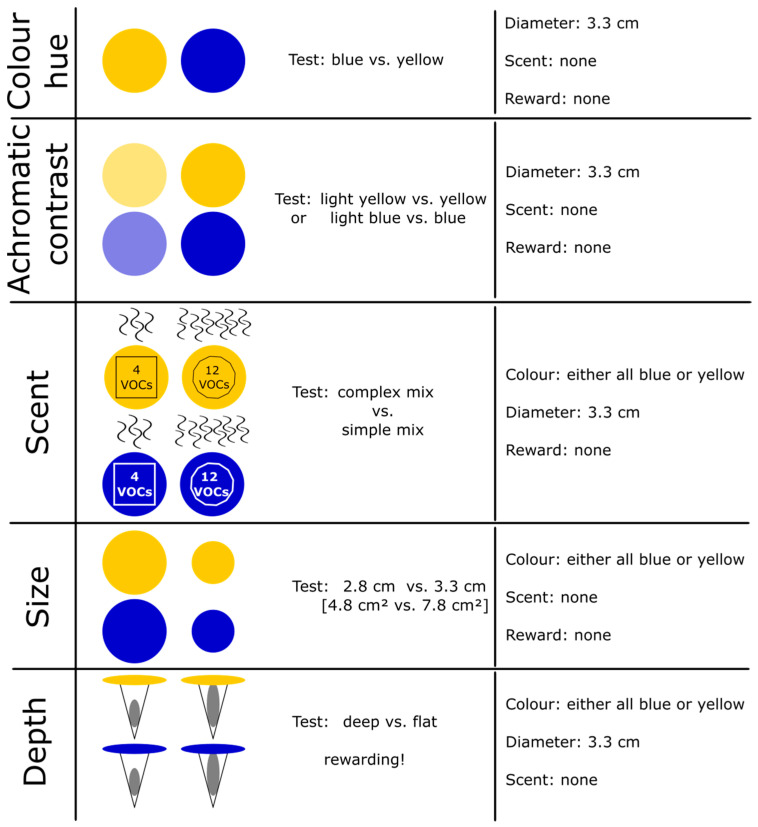
Overview of the different types of artificial flowers tested in the two-choice assays and their properties.

We performed preference tests with the common wild bee species *Lasioglossum villosulum* (Kirby 1902), *Bombus terrestris* (L. 1758), and *Osmia bicornis* (L. 1758) by using artificial flowers (Figure 1). The artificial flowers had a standardized design that was adapted to test for scent complexity, color hue, achromatic contrast, size, and flower depth (Figure 2). The flowers exhibited floral cues resembling those of *Anthemis tinctoria* (L.) (Figure 1C), a highly attractive flowering plant species for diverse wild bee species [49]. Behavioral experiments were performed as two-choice assays offering an artificial flower resembling floral cues of *A. tinctoria* against opposing floral cues. In addition, the synthetic mixture used for the scent complexity experiments was also employed to test antennal responses by means of electroantennographic detection (EAD).

### 2.1. Behavioral Assays

Behavioral experiments with all bee species were performed in the Botanical Garden of Ulm University when bee activity was high, between 8 am and 1 pm during the years 2022 and 2023. As the activity season differed among the species, the experimental time frame also differed for each species: experiments with *O. bicornis* were performed in April and May, with *B. terrestris* in June, and with *L. villosulum* in May and August. Experiments were undertaken when approximately 30 female bees of the relevant species were active in the flight cage. Only the first choice of a female bee that approached the test board was noted. Behavioral responses were recorded as approaches (targeted approximations to less than 5 cm) for all experiments. The experiments were repeated twice per test for each species, except in *L. villosulum* experiments, which were repeated three to four times because of low participation numbers.

Two colonies of *B. terrestris* bees were reared under standardized conditions [50] with founding queens descending from commercial colonies (Koppert Biological Systems, Netherlands). The nests were kept inside a portacabin (shaded, 25 °C, 60% humidity) attached to the flight cage (see details above). Due to the laboratory origin of the *B. terrestris* colonies, it was not possible to allow the bumblebees to freely forage outside of the cage. Pollen was provided inside the nest, whereas sugar water was offered in the flight cage and removed half an hour prior to experiments. During experiments, nest access was shut to control for the number of participating bees. The large number of bees enabled us to remove individuals that had participated in an experiment and thus to ensure that non-experienced individuals took part in the next experiment.

In the *O. bicornis* experiments, nesting aids and pupae were placed in a mesh flight cage (3.15 m × 2 m × 2.35 m, transparent acrylic glass roof, permanently attached to a portacabin). The pupae were divided into two batches, the first during mid-April and the second at 1–2 weeks subsequently in order to elongate the overall flight period. The performance of experiments was spread over the whole flight period to maximize the number of newly hatched individuals that could participate in an individual experiment, although some bees might have participated in more than one experiment. The mesh cage was opened before and after experiments to allow for foraging outside of the cage because the bees were not capable of adapting to feeders as a food resource in the flight cage and would rapidly starve. The bees nested in the provided nesting aids in the cage.

For *L. villosulum*, the set-up was adapted for ground-nesting bees by using a mobile flight cage (1.5 m × 1.5 m × 1.5 m Aerarium, Bioform, Nürnberg, Germany) placed above a nesting aggregation in the Botanical Garden of the University of Ulm. The cage was positioned on the ground for 20 min prior to experiments. The bees were allowed to freely forage outside of the cage before and after experiments because they were not capable of adapting to feeders as a food resource and would rapidly starve. Despite the position of the flight cage having been changed between the various experiments, some individuals might have participated in more than one experiment.

### 2.2. Artificial Flowers

The non-rewarding artificial flowers used for the choice experiments were made of a die-cut 3.3 cm diameter cardboard disk (300 g/m^2^, pre-colored cardboards: ‘Mango’ for yellow, ‘Zitrone’ for light yellow, ‘Ultrablau’ for blue, ‘Flieder’ for light blue; boesner GmbH, Germany) as the corolla and a lidless 1.5 mL Eppendorf tube as the floral tubes (Eppendorf SE, Hamburg, Germany).

Depending on the tested flower characteristics (Figure 2), three pairs of artificial flowers of one type were placed on a wooden board (ca. 50 cm × 50 cm) covered with green paper (130 g/cm^2^, pre-colored paper ‘Blattgrün’, boesner GmbH, Germany). The flowers were fixed into 1 cm deep holes by using dental wax (smiledental, Scheugroup, Germany). The distance within the pairs was 8 cm, and the distance across the pairs was at least 16 cm. The test board was placed at a height of approximately 40 cm at an angle of 45° facing the sun. During all experiments, the board was turned by 90° every 10 min to prevent the bees from learning the individual positions of the flowers and to change the alignment of the groups to one another and within one another. Artificial flowers were renewed every 20 min to avoid the behavior of the bees being influenced by conspecific scent marks [51].

The size and color of the corolla were adapted for each type of preference test (Figure 2). For the color hue experiments, the cardboard corolla hue was either blue or yellow, both of which had similar color contrasts to the background colors in the visual perception of the bees (details of color measurements and results given below). For achromatic contrast preferences, we tested colors of the same hues that differed in their achromatic contrasts, referred to as light blue vs. blue or a light yellow vs. yellow in this study. For scent complexity preference, either a complex scent mixture or one of three simple scent mixtures was applied on the corolla (details of synthetic mixtures given in Section 3.2). For flower size experiments, the artificial corolla diameter was either the standard 3.3 cm (7.8 cm^2^ surface, identical to the diameter of *A. tinctoria* flowers in personal observations) or a smaller variant with a diameter of 2.8 cm (4.8 cm^2^ surface; likewise die-cut) in blue or yellow.

### 2.3. Color Measurements

The cardboard corolla used in the behavioral experiments was blue, light blue, yellow, or light yellow in human visual perception. To estimate the visual perception of the bees, the spectral reflection of all cardboard structures of the experiment, including the green one used as a background, was recorded using an Ocean Optics Jaz Spectrometer (Ocean Optics, Inc., Dunedin, FL, USA). The wavelengths measured ranged from 300 to 700 nm and corresponded to the color spectrum as perceived by bees ([52], see Supplementary Material Figure S1). A deuterium-halogen lamp light was floated via a glass cable at an angle of 45 degrees. We used a black film canister as a black standard and a white standard from Ocean Optics. We then calculated the color perception for the bees by calculating the loci in the color hexagon after Chittka [53]. We used the receptor sensitivity data available for *A. mellifera*, as bee species typically do not differ substantially in their visual sensory system [52,54], and the standard daylight irradiance spectrum D65 [55] in the model.

Hexagon distances were calculated as Euclidean distances between the loci of the cardboard structures themselves and the locus of the uncolored hexagon center, with the measurement of the green cardboard being implemented as the background color. Achromatic contrast was defined as the distance between a color locus and the hexagon center, which resembled background coloration such as green leaf vegetation. Additional to the distance to the center, we calculated the angle within the hexagon. Thus, an imaginary vertical line from the center upwards was defined as 0°/360°. The yellow cardboard was chosen to resemble the color of *A. tinctoria* flowers and had a color angle of 146°. The blue cardboard showed almost maximum opposition and the corresponding light cardboard structures showed similar angles to their main colors.

### 2.4. Synthetic Scent Mixtures

Scent mixtures consisted of a total of 12 floral scent components of commonly occurring floral volatiles and substance classes identified in *A. tinctoria* and other flowering plant species [29]. The complex mixture contained all 12 VOCs, whereas the three simple mixtures contained 4 VOCs out of the 12, each with substance classes equally distributed across the mixes. All mixtures were adjusted to contain a total amount of 10^−5^ g substances mL^−1^ pentane with all substances added in equal volumes (total concentration: 10 µg mL^−1^, 2.5 µg mL^−1^ substance^−1^ in simple mixtures, 0.83 µg mL^−1^ substance^−1^ in the complex mixture; pentane: SupraSolv, Supelco, MilliporeSigma, Darmstadt, Germany). For scent preference experiments, an aliquot of 30 µL of the dilution (30 ng substance) was applied per artificial flower, and the experiments were started after a lapse of 15 min to allow for pentane evaporation. The complex mixture at a dilution of 1 µL of 10^−4^ g mL^−1^ (resulting in a total of 10 ng substance) was used in electrophysiological experiments.

### 2.5. Electrophysiological Experiments

To investigate whether the VOCs were perceived by the various bee species, we performed gas chromatography coupled with electroantennography (GC-EAD; [32]) and a scent mixture that contained all compounds that were also tested in behavioral experiments.

Each antenna was cut at its base and tip by using a micro-scalpel right immediately the experiments and was mounted between two capillaries containing insect Ringer’s solution (in mmol L^−1^: 137 NaCl, 5.4 KCl, 3.6 CaCl_2_). The required closed electric circuit was provided by gold wires connecting the capillaries. The GC-EAD set-up consisted of a gas chromatograph (GC; HP 6890, Hewlett-Packard, Agilent Technologies, Santa Clara, CA, USA) equipped with a flame ionization detector (FID) and it was coupled to an electroantennographic detection set-up (EAD; both Syntech, Hilversum, The Netherlands). Once the antenna was mounted, we injected 1 µL of the 10^−4^ g mL^−1^ complex mixture (resulting in a total of 10 ng substance) in the splitless mode at an oven temperature of 40 °C. After 1 min, the split opened, and the oven was heated at a rate of 10 °C min^−1^ to 250 °C (then held for 5 min). Hydrogen was used as the carrier gas at a constant flow of 2 mL min^−1^. We used a DB-5 column (length 30 m, inner diameter 0.25 mm, film thickness 0.25 µm, Agilent Technologies, Santa Clara, CA, USA) with an installed four-arm flow splitter (GRAPHPACK 3D/2, Gerstel, Mühlheim, Germany) that split the column at the end into two deactivated capillaries (length 50 cm, ID 0.32 mm), one leading to the detector and the other to the EAD set-up. Nitrogen was introduced as the make-up gas at 30 mL min^−1^ through the fourth arm of the splitter. The outlet of the EAD was placed in a cleaned and humidified airflow (glass tube, inner diameter 7 mm, air stream 100 mL min^−1^) directed over the prepared antenna.

In total, 10 antennae of *B. terrestris* and *O. bicornis* and 6 antennae of *L. villosulum*, all from different females, were tested. Antennal responses were recorded and analyzed using the software GcEad 2014 v1.2.5 (Syntech, Kirchzarten, Germany). The number of bee individuals responding to a compound were counted for each bee species, and compounds were considered to be active if they elicited a response in at least 50% of the tested antennae of a species.

### 2.6. Floral Depth Experiment with Rewarding Artificial Flowers

Additional experiments were performed with *O. bicornis* and *B. terrestris* to test for feeding preferences at different floral tube depths. Shallow artificial flowers resembled *A. tinctoria* flowers. The artificial flowers and the set-up were identical to the general set-up of the experiments, except that the flowers were rewarding. A medical-grade cellulose swab (Sugi^®^, Kettenbach GmbH & Co. KG, Eschenburg, Germany) either was placed inside the Eppendorf tube to fill the tube to its uppermost edge or was pushed to a depth of 0.5 cm into the tube (Figure 2). Sugar water (35%, fructose and glucose 1:1, Apiinvert, Südzucker AG, Mannheim, Germany) was added to the tubes to saturate the swabs. Depending on the position of the swab, bees were able to consume the sugar water either directly after landing on the corolla and without entering the floral tube (shallow flowers) or after crawling inside the flowers (0.5 cm deep flowers) to reach the reward. Due to the plain surface structure and ability to absorb liquids of the swab, the bees could only consume sugar water at the described positions inside the flowers. Behavioral responses of the bees were recorded as approaches (as defined above), landing (wingbeat stop), and drinking (crawling into the artificial flower and/or extending proboscis). The amount of sugar water consumed was determined by weighing the artificial flowers prior to an experiment, after 40 min and again after 2 h by using an accuracy weighing machine (accuracy 0.1 mg). This experiment was not performed with *L. villosulum* because the bees did not show efficient landing behavior on the artificial flowers for testing their feeding behavior.

### 2.7. Statistical Analyses

All statistics were performed in R Studio (version 2023.03.0, [56]). First, we built one binomial generalized linear model (GLM) per tested trait, resulting in a total of five global models. These models contained the data of all bee species, including bee species as a fixed factor to identify significant differences among the bee species. For the ‘achromatic contrast’ model, we added the base color (yellow or blue) as a random factor. For the ‘scent’ model, we added the mixture (mix 1, 2, or 3) as a random factor into our models. Significant differences between the bee species in these initial models resulted in more detailed models per trait test for each species. For a comparison of consumption rates, we performed Mann–Whitney U tests. Graphs were obtained using the ‘likert’ package [57].

## 3. Results

### 3.1. Preferences for Different Color and Size Properties

#### 3.1.1. Cardboard Properties and Model Flower Color

**Figure 3:**
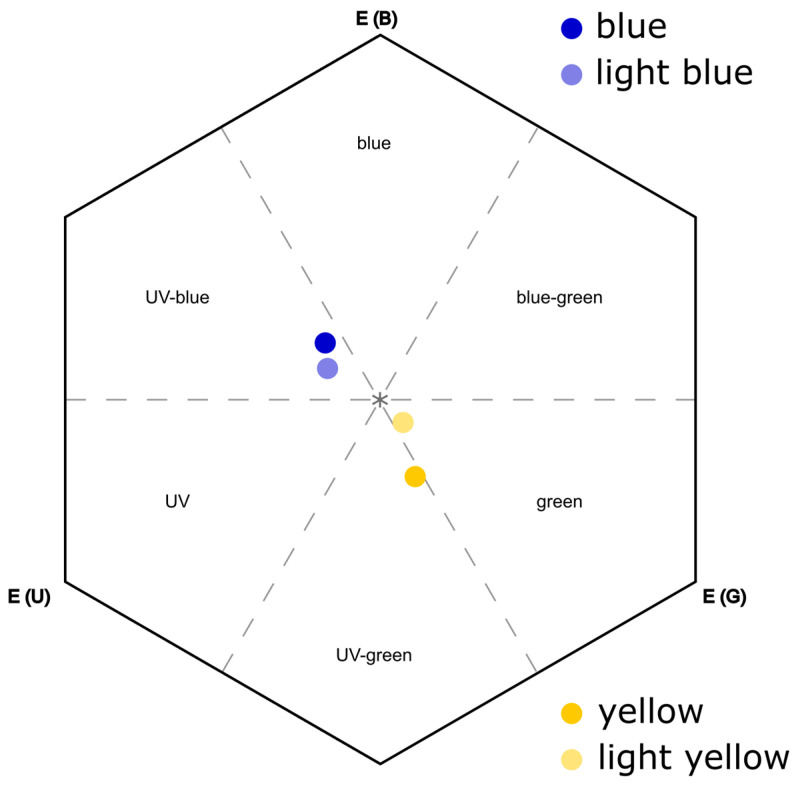
Bee color space showing the color loci of the cardboard structures used in the experiments in relation to the green cardboard used as a background color (asterisk in the middle). The color hexagon [53] is based on the sensitivities of the blue (B), green (G), and UV (U) bee receptors.

The exact colors of the cardboard structures were measured and re-calculated to the visual perception of bees against the green cardboard used as a background in the experiments (Figure 3). The intense colored cardboard structures showed an almost maximum opposition in the hexagon and were of a similar distance to the center, resulting in a similar achromatic contrast (blue: 0.217 units; yellow: 0.234 units). The corresponding light colors were of the same hue as the base colors but slightly differed in achromatic contrast (distance to center: light yellow: 0.091 units; light blue: 0.169 units).

#### 3.1.2. Color Hue Preferences

**Figure 4:**
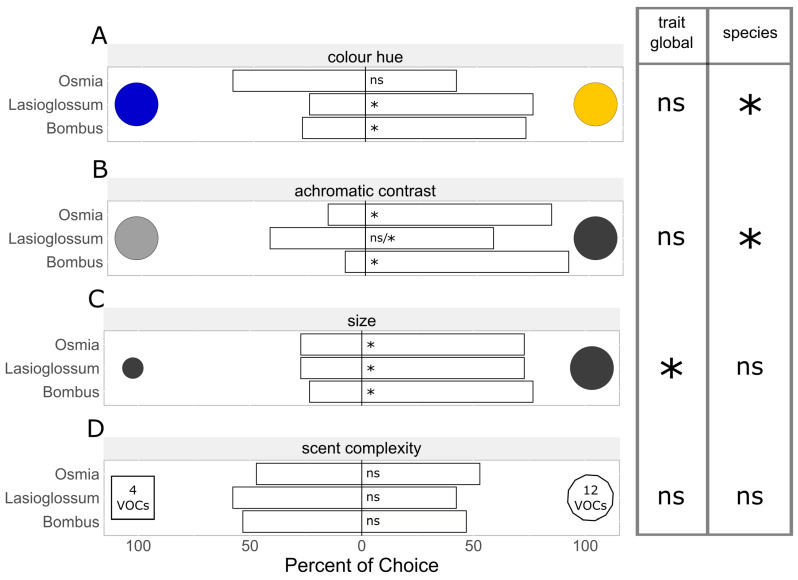
Preferences (percentage of choice) of the three bee species *Osmia bicornis*, *Lasioglossum villosulum*, and *Bombus terrestris* for the different floral cues in the two-choice assays: (**A**) color hue testing blue versus yellow, (**B**) achromatic contrast testing light versus more intense colors, (**C**) size testing small versus large corollas, and (**D**) scent complexity testing a simple synthetic mixture (4 VOCs) versus a complex one (12 VOCs). Artificial flowers were colored either yellow or blue in (**B**,**C**), and three different simple scent mixtures were used in (**D**) (see also Figure 2). Significant differences are indicated by asterisks (ns: *p* > 0.05; *: *p* < 0.05). Significances for global models (overall significance for the tested trait and significant differences between the species) are shown on the right, significances for floral traits within each species are indicated within the bars (ns/*: non-significant for blueish flowers, significant for yellowish flowers).

For hue preference, yellow vs. blue artificial flowers were tested (Figure 4, Table 1), with our results showing differences in hue preference among the bee species. *Lasioglossum villosulum* (*p* < 0.001) and *B. terrestris* (*p* < 0.001) both strongly preferred yellow over blue flowers. *Osmia bicornis* bees did not show a preference (*p* = 0.339).

#### 3.1.3. Achromatic Contrast Preferences

Achromatic contrast preference was tested by offering more intense colors with high achromatic contrast against lighter ones with low achromatic contrast (Figure 4, Table 1). Our results showed differences in achromatic contrast preference among the bee species (Figure 3). *Osmia bicornis* (*p* < 0.001) and *B. terrestris* (*p* < 0.001) both strongly preferred intensely colored over lighter colored flowers. For *L. villosulum*, we found no preference when analyzing all interactions (*p* = 0.227), but there was a preference for yellow over light-yellow artificial flowers (*p* = 0.0121).

#### 3.1.4. Size Preferences

Size preference was tested using two differently sized artificial flowers (Figure 4, Table 1). Larger artificial flowers were preferred across all bee species (*O. bicornis*: *p* = 0.028; *L. villosulum*: *p* = 0.004; *B. terrestris*: *p* < 0.001).

### 3.2. Preferences for Scent Complexity and Antennal Responses to Scents

#### 3.2.1. Scent Complexity Preferences

Preferences for the complexity of scent bouquets were tested using two visually identical but differently scented artificial flowers, with either a complex or a simple VOC mixture (Figure 4, Table 1). We did not observe a general preference of any of the species for the complex mixture over the simple mixtures (*O. bicornis*: *p* = 0.429; *L. villosulum*: *p* = 0.269; *B. terrestris*: *p* = 0.197).

#### 3.2.2. Antennal Responses to Scent Compounds

**Figure 5:**
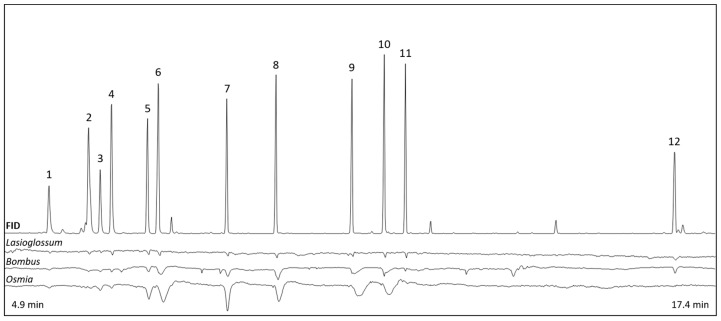
Examples of antennal responses (0.5 mV) of the bee species *L. villosulum*, *B. terrestris*, and *O. bicornis* to the compounds (FID: flame ionization detector of gas chromatography, 50 mV) used in the behavioral experiments testing preferences for scent complexity. Numbers of compounds correspond to numbers given in Table 2.

Most of the 12 tested VOCs were electrophysiologically active in all tested antennae of the wild bee species (Figure 5, Table 2). However, hexahydro farnesyl acetone in mixture 2 was only active in 50% and β-caryophyllene in mixture 3 was only active in 60% of the tested *O. bicornis* antennae. Another compound of mixture 2, eucalyptol, was only active in 40% of tested *B. terrestris* antennae. Only 66% of the *L. villosulum* antennae exhibited electrophysiological activity for myrcene in mixture 3.

### 3.3. Flower Depth Preferences

**Figure 6:**
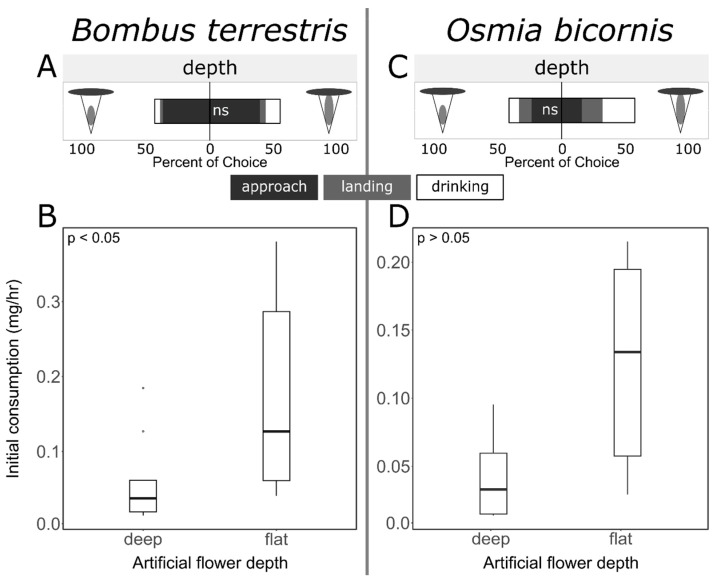
Preferences (percentage of choice) and consumption rates of *Bombus terrestris* (**A**,**B**) and *Osmia bicornis* (**C**,**D**). (**A**,**C**) Behavioral responses (a = approach, l = landing, d = drinking) to deep versus flat flowers for *B. terrestris* (**A**) and *O. bicornis* (**C**). (**B**,**D**) Consumption rates (mg/h) per artificial flower presented as a deep versus flat flower in *B. terrestris* (**C**) and *O. bicornis* (**D**) (Mann–Whitney U test, sample size: *Bombus:* 9 runs in 2 colonies, *Osmia:* 6 runs). Significances are indicated (ns: *p* > 0.05; *: *p* < 0.05).

Additional flower depth preference experiments were performed with *B. terrestris* and *O. bicornis* bees by using rewarding artificial flowers. Both bee species showed only a slight but not significant preference for flat flowers in the number of total approaches (bees that approached only or additionally showed landing and/or drinking behaviors) for flat or deep flowers (Figure 6; GLMs: *O. bicornis*: *p* = 0.088; *B. terrestris*: *p* = 0.097). This was also reflected by a trend for higher consumption from flat flowers by *O. bicornis* bees (Figure 6; Mann–Whitney U test, *O. bicornis*: *p* = 0.09), a trend that became significant after a 2 h feeding period (see Supplementary Material Figure S2). *Bombus terrestris* bees immediately exhibited a significantly higher consumption rate per flower (mg/h) from flat flowers than from deep flowers (Figure 6; Mann–Whitney U test, *p* = 0.011; for 2 h feeding period see Supplementary Material Figure S2).

## 4. Discussion

In this study, we gained insights into floral trait preferences of three wild bee species. The study increases the knowledge about non-model bee species that are important pollinators but whose floral cue preferences to find foraging plants were almost unknown so far. *Lasioglossum villosulum* belongs to the species-rich taxon of halictid bees, in which a wide range of different lifestyles is known from different species. Halictid bee species sociality can range from solitary to primitively eusocial, resulting in potentially high individuum abundances that contribute to pollination services in an area. *Osmia bicornis* has become a focus in agricultural management beside commercially used *B. terrestris* and *A. mellifera* pollinators in Germany and has been studied extensively regarding its biology and breeding (as reviewed by [58]). Regarding its actual interactions with flowers, it has been studied for its capability of perceiving colors and scent cues [25,26,54], but further insights were missing.

Our experiments showed consistent behaviors across the bees of the species *O. bicornis*, *L. villosulum*, and *B. terrestris* for most of the tested floral cues. All species clearly preferred larger flowers and mostly colors with a high achromatic contrast and none of the bee species preferred the complex synthetic scent mixture over a simple one containing a smaller number of volatiles. We also demonstrated different preferences among the species, particularly for color hues.

The differences found between species cannot be only explained by species-specific behaviors but are probably more a result of previous foraging experience. Bees quickly associate floral cues with rewards [13,59]. This means that floral cue preferences found in our study might change after foraging on other plant species which needs to be addressed in further studies with controlled foraging resources. Nevertheless, foraging-experienced bees still need the capability of switching host plants and identifying potential new ones based on distinct floral cues. This means that bees cannot only rely on previously encountered cues but depend on foraging strategies to explore potentially new host plants. Especially generalist bee species need to adapt to changing floral resources during their lifetime when the availability of flowering plants changes during the bees’ activity season. Experiments with flower-naïve individuals only allow conclusions on how bees rely on floral cues to find flowers on their very first flower visits but do not resemble natural situations after a few foraging flights. Cues that are used to identify new resources at first can decrease in relevance over time [60].

### 4.1. Preferences for Color Hue and Achromatic Contrast

Our experiments on hue preference showed the preference of *B. terrestris* and *L. villosulum* for yellow over blue flowers, whereas the same experiments gave inconclusive results regarding *O. bicornis* bees. Preferences for specific color properties are known to differ from the clade level down to the species level and also to depend on the visual detection capabilities of the species resulting in different pollination syndromes [39]. In general, many insects are known to rely on visual cues, such as color, to find and identify host plants. Yellow preferences have been observed, for example, in the hoverfly *Episyrphus balteatus* [61,62], and a UV-blue preference is exhibited by the stingless bee *Melipona mondury* [63]. Melittophilic flowers seem to adapt to the visual system of bees by optimizing their patterns to maximum detectability, e.g., by means of a high achromatic contrast between floral and background colors detected by the green receptor in the bee eye [64]. A high attractiveness of intense colors has also been demonstrated by our findings showing that *B. terrestris* and *O. bicornis* bees generally prefer artificial flowers with high achromatic contrasts, whereas *L. villosulum* prefer yellowish flowers. This distribution of preferences also agrees with in-field observations indicating that the preferences of different bee species fit the distribution of floral colors: the more common a color is, the more species visit it [65].

Both *O. bicornis* and *L. villosulum* bees were foraging-experienced as we allowed them to forage outside the flight cages between trials. All experiments were performed in flight cages located in the Botanical Garden in Ulm, which offers diverse plant communities, including many potential host plants for wild bees. *Osmia bicornis* is a broad generalist species [66], and when the experiments were performed, a large variety of potential host plants of different colors was blooming in the surroundings (e.g., *Ranunculus* spp., orchard trees, *Muscari* spp.). Therefore, we assume that the bees visited differently colored flowers while foraging outside of the flight cage. They probably learned to associate different floral colors with a reward and were attracted by both yellow and blue colors in our experiment for this reason. Visiting a wide range of flowers, they had probably also encountered flowers of low and high achromatic contrast. However, in our experiments, *O. bicornis* clearly preferred only high contrasting colors. The results of the behavioral experiment agree with those in the literature describing the greater attraction of more highly contrasting colors for bees [3,39]

*Lasioglossum villosulum* is described as a polylectic species, visiting different plant taxa, but the bees show strong preferences for Asteraceae and are considered to be broadly oligolectic [66,67]. When our experiments were performed, the host plant *Crepis biennis* (Asteraceae) was in bloom, and we observed bees visiting these flowers. Moreover, all bee individuals carried yellow pollen loads when coming back to their nests. The strong preference of these bees for naturally occurring yellow flowers possibly explains our finding of their preference for yellow over blue artificial flowers. A similar preference of yellow color cues has already been shown for *L. lanarium* females in Australia, while another unspecified Australian *Lasioglossum* species remained inconclusive [20]. As the *L. villosulum* bees of our study did not, or at least not to a large extent, forage on blue flowers, they were probably not attracted by blue-colored flowers in our experiments and did not show a preference when different blueish colors were presented. In contrast, they clearly preferred yellow flowers with high achromatic contrast over light-yellow flowers, which probably resembled their foraging behavior in the field. None of the plants with blue flowers that bloomed during the experiments, namely mostly *Salvia pratensis* and *Knautia arvensis*, were visited by *L. villosulum* bees [66]. Strong color preferences for the floral colors of their host plants have also been described for highly specialized bee species. For example, *Hoplitis adunca* females prefer the blueish colors of their *Echium* host flowers [16]. The color preference found in *H. adunca* is pronounced even in naïve individuals that have not yet encountered their host plants and remains constant in flower-experienced individuals. In specialized *Macropis* bees, visual cues become more important with foraging experience compared to naïve bees [68]. In this, it would be interesting to test whether the yellow preference in *L. villosulum* bees is a learned behavior or inherent.

In contrast to the other two bee species, only *B. terrestris* were unable to forage freely outside in between trials, and thus, they were inexperienced visiting natural flowers but experienced with regard to artificial feeders in the flight cage. *Bombus terrestris* is, like *Apis mellifera*, one of the best studied bee species regarding their color preference and perception. For a long time, blue was considered as the preferred color of bumblebees following Müllers’ observations in alpine areas [69], but yellow preferences were found in other regions [70]. The contrast between a floral color against the background colors such as green leaves can be even more important than the color hue itself [39,40]. Flowers with higher spectral purity are even preferred, when *B. terrestris* bumblebees are priorly trained to other colors [71]. In our experiments, *B. terrestris* bees were not trained prior to testing. However, they did prefer yellow over blue artificial flowers, a result that might be explained by the slightly higher contrast of yellow artificial flowers against the provided green background.

### 4.2. Preferences for Large Corollas

All tested bee species clearly preferred larger artificial flowers over smaller ones in our experiments. Similar to increased achromatic contrast, larger flowers or larger floral display size (e.g., higher numbers of racemes per stem) can be detected more easily by an approaching bee, resulting in a higher attractiveness of larger flowers [7,8]. The size of flowers also determines whether bees can perceive the specific color hue of a flower; with flowers below a certain size (depending on the distance between the flower and an approaching bee), the bees detect only the achromatic contrast that a flower forms towards background colors [72]. In addition to detectability, floral area is directly correlated to the availability of floral rewards in the form of pollen or nectar [73]. An increased floral area has also been shown to be correlated with visitation rates of *Bombus* and non-*Bombus* wild bees in the field [74]. It was also demonstrated that *B. terrestris*, *B. lucorum*, *and B. pascuorum* initially choose flowers with larger display sizes when first interacting with an unknown plant species [60]. These findings are in line with our results of choosing larger corollas of artificial flowers, which we now demonstrate also for *L. villosulum* and *O. bicornis* bees in addition to the well-studied *Bombus* species. However, not all former studies consistently describe preferences for larger flowers in bees and other insect species. In a laboratory experiment, *B. terrestris* did not exhibit an innate preference for large flowers but rather a learned association of large flowers with increased rewards [75]. Furthermore, *Megachile rotundata* bees, for example, prefer an intermediate display size in field experiments [7] and *Episyrphus balteatus* hoverflies seem to prefer smaller flowers [61].

### 4.3. Responses to Scent Complexity

The tested bee species were presented with VOCs in three synthetic mixtures that were presented against an overall mixture with higher complexity (number of VOCs). We tested for preferences in scent complexity because natural scent bouquets contain various volatiles that address and balance out a multitude of functions, including the attraction of agonists and repulsion of antagonists [37]. However, olfactory information or the function of relevant components probably need to be learned by a potential pollinator and associated with a specific reward or flowering plant species [76] and scent complexity does not seem to be important per se. These findings are supported by the fact that honeybees can be trained to a variety of volatiles that are not necessarily plant- or reward-specific [77]. Conversely, plants benefit from being recognized when floral cues are learnt by a pollinator, as the re-visiting of flowers of the same species increases pollination success. Despite the costs involved, a plant investing in more distinguishable floral cues will thus achieve better pollination [38].

All bee species tested in our experiments had had some contact with floral scents prior to the experiments but were not trained to the offered synthetic mixtures. The *O. bicornis* and *L. villosulum* bees were foraging-experienced and collected pollen to provision their nests before and in between experiments. The *B. terrestris* bees, on the contrary, did not forage on flowers but adults and larvae had contact with the pollen offered within their colony. Pollen is known to emit not only pollen-specific volatiles but also scents of the floral tissue [78]. Even before visiting flowers, bee larvae are in contact with scents, as pollen can be advertised by a flower using VOCs [79]. However, in experiments with *O. bicornis*, it could not be assured nor ruled out that the flower selection of adult individuals is driven by food provision to the larval stage [80].

While foraging on flowers, bees associate floral scent cues with rewards [13,81,82]. In particular, information regarding the reward and fitness status of a flower can be crucial for a pollinator, as bees try to optimize foraging and consequently will avoid visiting unrewarding flowers [83]. For example, *Osmia* sp. bees rely on olfactory cues to detect nectar and are able to differentiate between rewarding and unrewarding flowers [84].

Despite our inconclusive results, preferences for scent complexity need to be studied further, for example, by the manipulation of scent bouquets in a specific system or by the analysis of single compounds to determine their specific functions [76]. For example, *H. adunca* bees recognize their host plants via the key compound benzoquinone when they are on their first search for host plants. Foraging-experienced females, on the other hand, are no-longer attracted by this single compound but rely on a complex bouquet [16,85]. In our experiments, we prepared a bouquet that resembled that of *A. tinctoria* but also used a limited number of volatiles emitted by *A. tinctoria* and added further typical floral volatiles to prepare standardized mixtures with widely distributed scent compounds, potentially leaving out volatiles important for the recognition of or for achieving high attraction to *A. tinctoria*.

Our EADs showed that most VOCs elicited antennal responses in all species, and only a few ones differed across the species, e.g., the monoterpene β-myrcene or the sesquiterpene hexahydro farnesyl acetone. In a previous study on electrophysiological activity in the antennae of *O. bicornis* bees, Klatt et al. [26] identified varying sensitivities to compounds emitted by strawberry flowers. In a following study on strawberry scent by Cordeiro and Dötterl [25], two of our study species *B. terrestris* and *O. bicornis* responded to the same VOCs in electroantennographic experiments and were attracted by the scent bouquet. Similar to this study, electrophysiologically active compounds need to be identified in the natural scent of *A. tinctoria* and the attractiveness should be tested in future experiments.

Our experiments lead us to assume that the selected volatiles and bouquets did not reflect important scent information that the bees recognize during their foraging flights and that the complexity of the floral scent bouquet per se, if it does not resemble a natural system, plays a minor role.

### 4.4. Bees Prefer Low-Effort Nectar Exploitation

In the flower-depth experiments, *O. bicornis* and *B. terrestris* consumed significantly more sugar water from flat flowers than from deep flowers. Normally, visitation rates or flower choices by pollinators can be explained by flower depth and tongue length: to efficiently exploit a flower’s nectar, the length of the tongue of the bee needs to match the flower depth [44,45,86]. This contrasts with our results, as the *O. bicornis* and *B. terrestris* bees did not prefer deep flowers, despite both species having relatively long tongues. In nature, the opening of bilabiate flowers with deep spurs are mostly facing vertically, resulting in a different approaching angle of the bee: bees rather approach such flowers from the side and not from above [87]. Thus, our artificial flowers might have been more similar to flat flowers in the visual perception of the bees, although we presented the board with flowers at a slight angle. Further, spur depth often coinheres with flower complexity and especially *B. terrestris* is known to efficiently handle morphologically complex flowers with deep nectar spurs because of their large body size [88].

The reward quantity of the artificial flowers did not reflect natural plant communities with competing floral visitors and a limited availability of rewards. In contrast, the artificial flowers offered unlimited amounts of sugar resources without competing floral visitors. According to optimal foraging theories, bees will choose flowers with the best energy output resulting from the sum of the energy cost for the handling of flowers versus the obtained reward [83]. The handling of flowers requires a high learning capacity, and bees are limited in the number of different morphologies they can optimally handle [5]. Furthermore, some morphological barriers are energy-costly, for example, with respect to force needed to widen a flower entrance to reach the nectar as is the case in *Salvia* or *Antirrhinum* flowers [89]. In our case, a bee crawling into a flower would have expended more energy compared with a bee simply landing on the cardboard corolla, as bees could easily land on the corolla of the flat flowers and reach the unlimited reward by merely extending its proboscis. Our bees rapidly learned that they required more effort to explore the deep flowers to collect the same resource. In consequence, the bees preferred flat flowers because of the higher energy incomes in the non-competing array of artificial flowers.

In natural bee–plant communities, bees visit flowers with complex morphologies and hidden rewards to avoid competition with other bees on highly visited flowers [90]. In comparison, highly visited flowers that are visited by a wide range of species are often flat flowers that openly present their rewards and are easy to exploit. Thus, these plant species can compensate for their open morphology by increasing the relative amount of long-chain sugars, thereby decreasing evaporation [91], or can restrict access by producing toxins in their rewards [92,93,94]. However, in our study, neither competition with other species nor a difference in reward quality was present, and we therefore explain our results on the basis of the bees choosing flowers with the best energy output.

## 5. Conclusions

In this study, we gained knowledge about the floral cues influencing the flower choices of three German wild bee species, two of which were severely under-studied so far. While floral cue preferences have been extensively studied in the common and agriculturally used bee species *A. mellifera* and *B. terrestris*, studies on non-model organisms are still largely lacking. The importance of solitary or non-eusocial generalist bee species in pollination has been acknowledged, but we still need to gain a further understanding of the attractants in comparative studies. We have found a subset of cues that are commonly attractive across the tested species. Intensively colored flowers and large floral displays seem to be important cues for attracting generalist bees. The total number of floral volatiles had a subordinate function in attracting bees that were not experienced with the presented scent bouquet in previous foraging flights. Differences in behaviors among species not only reflect the ecological context of the species such as host–plant preferences but also depend on the foraging experience of bee individuals.

## Figures and Tables

**Table 1:** Detailed statistics of binomial GLMs. For each test and species, the used data subset (‘all’ refers to the species-specific subset) and the significantly preferred floral trait are given (‘non’ indicates non-significant results).

Test	Species	Data	Preferred	Estimated	Std. Error	z-Value	*p*-Value
hue	*L. villosulum*	all	yellow	1.099	0.320	3.430	<0.001
	*B. terrestris*	all	yellow	0.937	0.155	6.047	<0.001
	*O. bicornis*	all	no preference	−0.375	0.392	−0.957	0.339
size	*L. villosulum*	all	large	−0.981	0.229	−2.898	0.004
	*B. terrestris*	all	large	−1.187	0.269	−4.409	<0.001
	*O. bicornis*	all	large	−0.833	0.379	−2.199	0.028
achromatic contrast	*O. bicornis*	all	high contrast	−1.609	0.447	−3.599	<0.001
	*B. terrestris*	all	high contrast	−2.311	0.303	−7.635	<0.001
	*L. villosulum*	all	non	−0.296	0.245	−1.208	0.227
		yellow vs. light yellow	high contrast	−0.981	0.391	−2.509	0.0121
scent	*O. bicornis*	all	non	−0.114	0.144	−0.791	0.429
	*B. terrestris*	all	non	0.128	0.099	1.289	0.197
	*L. villosulum*	all	non	0.310	0.281	1.105	0.269

**Table 2:** Substances tested in behavioral and electrophysiological experiments. The complex mixture contained all given substances, whereas the three different simple mixtures (mix 1–3) contained 4 VOCs each. The percentage number of electrophysiological responses to each compound is given for *Osmia bicornis*, *Bombus terrestris*, and *Lasioglossum villosulum*. The chemical class and the retention index (RI) of the substances are also given.

No. #	Substance	RI	Substance Class	Mix	Electrophysiological Responses		
					*O. bicornis* (*n* = 10)	*B. terrestris* (*n* = 10)	*L. villosulum* (*n* = 6)
1	β-myrcene	986	monoterpene	3	80%	80%	66%
2	eucalyptol	1032	monoterpene	2	70%	40%	100%
3	(*E*)-β-ocimene	1045	monoterpene	1	100%	100%	100%
4	γ-terpinene	1058	monoterpene	2	100%	90%	100%
5	β-linalool	1099	monoterpene	1	100%	100%	100%
6	2-phenylethanol	1112	benzenoid	2	100%	100%	100%
7	methyl salicylate	1194	benzenoid	3	100%	100%	100%
8	2-phenethyl acetate	1255	benzenoid	1	100%	100%	100%
9	eugenol	1353	benzenoid	3	100%	100%	100%
10	benzyl isovalerate	1396	benzenoid	1	100%	90%	100%
11	β-caryophyllene	1426	sesquiterpene	3	60%	80%	100%
12	hexahydro farnesyl acetone	1840	sesquiterpene	2	50%	90%	100%

## Data Availability

The raw data supporting the conclusions of this article will be made available by the authors on request.

## Supplementary Materials

The following supporting information can be downloaded at https://www.mdpi.com/article/10.3390/insects15060427/s1, Figure S1: Reflectance spectra of the cardboards tested in our experiments. Figure S2: Consumption in feeding experiment after 2 h.
