# Supplementary material for: Floral Trait Preferences of Three Common wild Bee Species
Source: Insects. 2024 Jun 6;15(6):427. doi: 10.3390/insects15060427 (PMC11203783; doi:10.3390/insects15060427)
Supplement: Supplementary file 1 [file insects-15-00427-s001.zip › insects-3007809-supplementary.pdf]

insects

Supplementary Material

Floral trait preferences of three common wild bee species

Kim C. Heuel, Tim A. Haßlberger, Manfred Ayasse and Hannah Burger

Supplementary Figure S1

The cardboard corolla used in the behavioral experiments was blue, light-blue, yellow, or light-yellow in human visual perception. The spectral reflection of all cardboards as well as the green background paper was recorded using an Ocean Optics Jaz Spectrometer (Ocean Optics, Inc., Dunedin, FL, USA). The wavelengths measured ranged from 300 to 700 nm and corresponded to the color spectrum as perceived by bees [1]. A deuterium-halogen lamp floated light via a glass cable at an angle of 45 degrees. We used a black film canister as a black standard and a white standard from Ocean Optics.

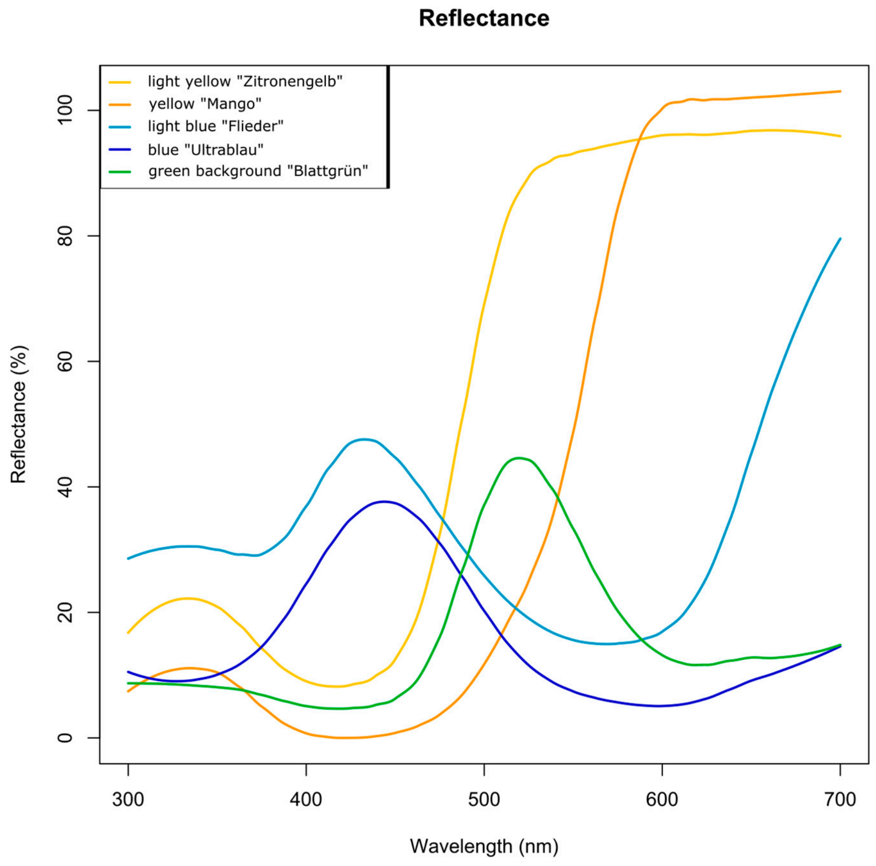

Figure S1: Spectral reflection of cardboards used for artificial flowers, including the green used as background.

Supplementary Figure S2

The amount of sugar water consumed was determined by weighing the artificial flowers prior to an experiment, after 40 min and again after 2 hr by using an accuracy weighing machine (accuracy 0.1 mg).

We found a significant difference for higher consumption from flat flowers in both *B. terrestris* and *O. bicornis* bees after 2 hrs. For initial approaches and consumption after 40 min see main manuscript.

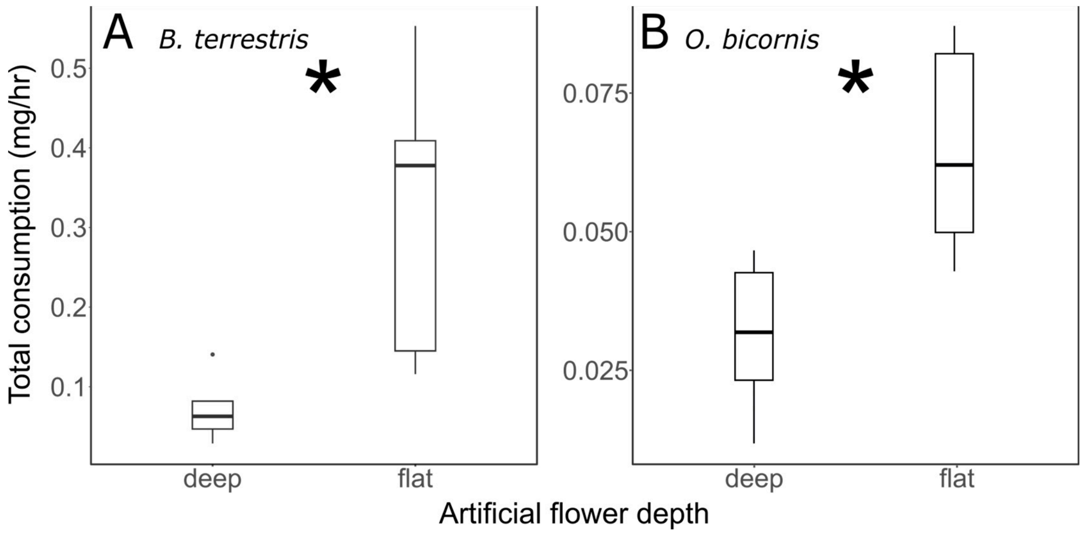

Figure S2: Consumption rates (mg/h) per artificial flower presented as a deep versus flat flower in B. terrestris (A) and O. bicornis (B) (Mann-Whitney U test, sample size: Bombus: 9 runs in 2 colonies, Osmia: 6 runs). Significances are indicated (: p<0.05).*

References

1. Peitsch, D.; Fietz, A.; Hertel, H.; Souza, J. de; Ventura, D.F.; Menzel, R. The spectral input systems of hymenopteran insects and their receptor-based colour vision. *J. Comp. Physiol. A* **1992**, *170*, 23–40.
